# A multivariate approach to explore the volatolomic and sensory profiles of craft Italian Grape Ale beers produced with novel *Saccharomyces cerevisiae* strains

**DOI:** 10.3389/fmicb.2023.1234884

**Published:** 2023-07-27

**Authors:** Rocchina Pietrafesa, Gabriella Siesto, Maria Tufariello, Lorenzo Palombi, Antonietta Baiano, Carmela Gerardi, Ada Braghieri, Francesco Genovese, Francesco Grieco, Angela Capece

**Affiliations:** ^1^Scuola di Scienze Agrarie, Forestali, Alimentari ed Ambientali, Università degli Studi della Basilicata, Potenza, Italy; ^2^Spinoff StarFInn S.r.l.s., Scuola di Scienze Agrarie, Forestali, Alimentari ed Ambientali, Università degli Studi della Basilicata, Potenza, Italy; ^3^Consiglio Nazionale delle Ricerche, Istituto di Scienze delle Produzioni Alimentari (ISPA), Lecce, Italy; ^4^Consiglio Nazionale delle Ricerche, Istituto di Fisica Applicata “Nello Carrara”, Firenze, Italy; ^5^Dipartimento di Scienze Agrarie, degli Alimenti e dell’Ambiente, Università di Foggia, Foggia, Italy

**Keywords:** fruit beer, indigenous strains, volatile organic compounds, sensory analysis, multivariate statistical analysis

## Abstract

This study investigated the influence of three *Saccharomyces cerevisiae* strains, selected from different matrices - CHE-3 (cherry), P4 (sourdough) and TA4-10 (grape must) - on characteristics of Italian Grape Ale (IGA) beers obtained at microbrewery scale. A multidisciplinary approach, combining results from analysis of chemical, volatile and organoleptic profiles of the beers, was adopted to underline the relationships between yeast starter and the quality of final products. Detection volatile organic compounds (VOCs) by Gas-Chromatography coupled with Mass Spectrometry (GC-MS) after extraction carried out by head-space micro-extraction (HS-SPME) revealed that the beer obtained by P4 strain differed from the others for its higher concentrations of esters, alcohols, and terpenes as confirmed by PCA (principal component analysis) and Cluster heatmap. Furthermore, sensorial analysis and consumer test showed that this sample differed from others by more pronounced notes of “fruity smell and floral” and “olfactory finesse,” and it was the most appreciated beer for smell, taste, and overall quality. Conversely, CHE-3 was the sample with the lowest concentrations of the identified volatiles and, together TA4-10, showed the highest scores for smoked, yeast, malt, and hop notes. As far as we know, these are the first results on the application of indigenous *S. cerevisiae* strains in the production of craft IGA beers analyzed through a complex multivariate approach.

## 1. Introduction

The Beer Judges Certification Program (BJCP) recently recognized a new subcategory of special fruit beers, denoted as Italian Grape Ale (IGA; BJCP, 2015). Produced primarily by craft breweries, this new style of beer joins two key products of the Italian beverage industry, i.e., beer and wine. IGA beers are produced with the addition of grapes or must or winemaking waste in a range from 5 to 40% of the total weight of the wort. The must addition significantly influences the sensory profile, thus offering to the brewers the opportunity to diversify their production, through the obtainment of beers characterized by a complex sensorial profile (Castro Marin et al., 2021). Indeed, an increase of phenolic acids and volatile molecules was detected in IGAs produced by adding must from grapes of the cv Lambrusco (Castro Marin et al., 2021). IGAs belong to the group of ale beers and are obtained from the fermentation process driven by strains belonging to the *Saccharomyces cerevisiae* species. Esters, alcohols, acids, aldehydes, ketones, hydrocarbons, sulfur compounds and volatile phenols are the main volatiles detected in beer, they being able to influence their aroma and flavor both individually and in a synergistic or antagonistic way (Pinho et al., 2006). Different strains of *S. cerevisiae* can produce significantly different flavor profiles when fermenting the same substrate and this is a consequence of two factors: the differential ability of yeast strains to release varietal volatile compounds from their non-volatile precursors, and the differential ability to *de novo* synthesize yeast-derived volatile compounds (Vilanova and Sieiro, 2006).

The use of pure yeast cultures, pioneered by Christian Hansen in the 1880s, greatly improved the consistency and quality of beer. Moreover, the increasing popularity of both the craft beer market (Garavaglia and Swinnen, 2018) and the consumer interest in new beer styles (Aquilani et al., 2015; Romano et al., 2023) have stimulated the research for new brewing yeasts (Gibson et al., 2017; Hittinger et al., 2018). Nowadays, different strategies are available for providing new starter cultures, such as evolution, mutagenesis, breeding, and yeast isolation from various environmental niches (Steensels et al., 2014a,b; Berbegal et al., 2019). The latter option represents one of the most attractive tools for the obtainment of alternative yeasts with brewing potential as it takes advantage of the natural biodiversity of the microorganisms found within specific geographical regions, which can incorporate novel flavor and aroma compounds into the fermented products. In fact, the use of locally selected yeast strains with strain-specific metabolic characteristics could positively affect the final quality of fermented beverage (Capece et al., 2010; Berbegal et al., 2018) and ensure the maintenance of the typical sensory properties of products deriving from any given region (Valles et al., 2008; De Simone et al., 2021). In the last years, yeast research has turned toward the use of food substrates that represent a potentially valuable source of strains with beneficial characteristics such as an increased resistance or new flavor and aroma profiles, which could be applied in a controlled manner to industrial brewing systems (Cubillos et al., 2019). Yeasts potentially useful for brewing have been isolated from cacao, kombucha, and sourdough cultures (Mascia et al., 2015; Bellut et al., 2018; Holt et al., 2018). With this in mind, in order to improve the quality of new craft beer styles, the producers have sought not only to diversify the choice of raw materials, but also by applying the microbial cross-over, which is the use of new yeast starter cultures, usually employed in other fermentation process (Dank et al., 2021).

In line with this strategy, three indigenous *S. cerevisiae* selected from different matrices, CHE-3 (cherry), P4 (sourdough) and TA4-10 (grape must), were recently applied in brewing trials for IGA production at a laboratory scale; these selected yeast strains resulted potentially able for differentiation and quality improvement of IGA beer production (Siesto et al., 2023). In the present study, these indigenous strains were tested during inoculated fermentations at microbrewery scale. The strain influence on chemical, aromatic and sensory profiles of final products was evaluated through a chemometric approach (principal component analysis, Cluster Heatmap, Partial least square-correlation) in order to validate these strains as novel starters for IGA production. To the best of our knowledge, this investigation is the first one concerning the application of the microbial crossover for the production of craft IGA beer in Southern Italy.

## 2. Materials and methods

### 2.1. Yeast starters

In this study, four *Saccharomyces cerevisiae* strains, already tested during brewing trials at laboratory scale (Siesto et al., 2023) were used. Three strains (CHE-3, P4 and TA4-10) were indigenous yeasts, previously isolated from different foods and belonging to the UNIBAS Yeast Collection (UBYC), University of Basilicata (Potenza, Italy), whereas the commercial starter SafAle US-05 (US-05)—American top-fermenting yeast (Fermentis, Marq en Baroeul, France) was used as reference strain. The yeasts were cultured aerobically in Erlenmeyer flasks containing 2 liters of YPD medium (yeast extract 10 g/L, peptone 20 g/L, dextrose 20 g/L) and incubated for 24 h at 20°C under stirring at 180 rpm, using a digital orbital shaker. These cultures were used as pre-inoculum for biomass production for micro brewing trials, following the procedure previously described (Capece et al., 2011). Briefly, the pre-cultures were inoculated in a vessel containing 4.5 L of YPD liquid by using the BioFlo/CelliGen 110 bioreactor (Eppendorf, Germany), with the following growth parameters: temperature at 20°C; stirring at 400 rpm; oxygen at 4 vvm. After 24 h, the biomass was recovered by centrifugation carried out at 5000 rpm for 10 min at 4°C; the recovered cell pellets were washed with saline solution (0.9% NaCl) and maintained at 4°C until their use. The cell density of biomass was assessed by viable cell count on YPD medium.

### 2.2. Beer production

The four strains were tested in a micro brewing plant (Birrificio Sorrento, Sorrento, Italy). Ten hectoliters of wort, prepared by using the commercial extract malt for Pale Ale beer (MrMalt^®^, Udine, Italy) already tested during laboratory scale trials (Siesto et al., 2023); the wort was boiled for 20 min with hop of Cascade variety. At the end of boiling, the beer wort was blended with 15% of frozen grape must (Falanghina variety). This percentage was chosen as it gave the best results during laboratory-scale trials and it was the most frequently used by this producer. The obtained wort, characterized by a content of total soluble solids of 11.4° Plato (corresponding to an original gravity of 1.044), was inoculated with each starter at inoculum level of 1 × 10^7^ CFU/mL.

The analysis of viable cells in fermentation wort was assayed by plate counting on Wallerstein Laboratory Nutrient Agar medium (WL, Oxoid, Hampshire, UK), a yeast differential culture medium (Pallmann et al., 2001).

The primary fermentation lasted 10 days, and then the samples were transferred into other steel tanks for the maturation step, performed at low temperatures (10°C) for 1 month. The secondary fermentation was carried out by disposing the samples in 500 mL bottles in the presence of 5 g/L of sucrose for 2 weeks at 10°C. At the end of secondary fermentation, the samples were maintained at 4°C for 2 months in order to allow flavor maturation and stability. Afterward, the beers were evaluated for analytical and aromatic composition.

During fermentation, the starter evolution was monitored by yeast viable cell counts on WL nutrient agar medium (Wallerstein Laboratory; Oxoid, Hampshire, UK) and by plate incubation at 26°C for 5 days. In details, the following sampling points were considered: after 24 h of inoculation, at the end of primary and secondary fermentations. For each isolation time, the plates containing a statistically representative number of colonies were counted and around 20 colonies were purified on YPD plates for identification by 5.8S ITS-RFLP analysis.

### 2.3. Standard quality attributes

The content of total soluble solids (°Plato) was determined by using a refractometer (Hanna Instruments, Padua, Italy). For bitterness determination, craft beers were decarbonated and 10 mL of samples were submitted to extraction of bitter substances by using 1 mL of hydrochloric acid 3M and 20 mL of pure iso-octane (Popescu et al., 2013). After that, the samples were shaken vigorously for 5 min at room temperature and centrifuged for 15 min at 4000 rpm. The iso-octane phase was decanted and drained, whereas the sample tube was maintained in dark for at least 30 min before measuring the absorption at 275 nm by a spectrophotometer (SPECTRO Star Nano, BGM Labtech). For calculation of bitterness, expressed as International Bittering Units (IBU), the average values of three determinations were used and the following equation was applied:

IBU = 50 × A275

where A275 correspond to the absorbance at 275 nm.

The beer color, was expressed as European Brewing Convention (EBC) units, determined by a colorimeter (SA-130, S.A.M.A. Italia S.r.l., Viareggio, Italy) and calculated as follows:

EBC color: 25.5 × (A430 -A700)

where A430 and A700 correspond to the absorbance at 430 and 700 nm, respectively.

The turbidity was measured in a turbidimeter (TL23, Hach^®^, Loveland, Colorado, US) and expressed in nephelometric turbidity units (NTU).

### 2.4. HPLC analysis

Organic acids were identified using an Agilent Hi-Plex H column (300 × 7.7 mm; 8.0 μm internal particles; Agilent Technologies, Santa Clara, CA, USA). The temperature of the column compartment was maintained at 70°C. The flow rate applied was 0.4 mL/min with a run time of 30 min. The phase was represented by 4.0 mM/L H_2_SO_4_ in ultrapure water. Standard solutions were injected to obtain the retention time for each compound. A Diode Array Detector settled at 210 nm was used.

The concentrations of maltodextrin, sucrose, maltose, maltotriose, glucose, fructose, glycerol, and ethanol were quantified using the same type of column used for organic acids. The mobile phase was represented by deionized water and a constant flow rate of 0.6 mL/min. A run time of 30 min was applied. Sugar detection was carried out through a Refractive Index Detector (RID). Quantification of individual organic acids and sugars was performed directly by the Chem-Station software (Agilent) using a five-point regression curve (R^2^ ≥ 0.99) based on authentic standards.

### 2.5. Volatolomic profile of IGA beers

Acetaldehyde, ethyl acetate, acetoin, n-propanol, isobutanol, n-butanol, 2-methyl-1-butanol and 3-methyl-1-butanol, were quantified by direct injection of 1 mL of beer samples into a packed glass column (80/120 Carbopak B/5% Carbowax 20 M, Supelco, Sigma-Aldrich, Milano, Italy) by using an Agilent 7890A Gas-Chromatograph, following the protocol previously reported (Capece et al., 2021).

Volatile organic compounds (VOCs) were identified and quantified by Gas-Chromatography coupled with Mass Spectrometry (GC-MS) after extraction carried out by head-space micro-extraction (HS-SPME) according to Palombi et al. (2023). Briefly, 100 μL of internal standard solution (ISTD, 4-methyl-2-pentanol, 300 mg/L) was added to a volume of 5 mL of beer in a 20 mL headspace vial (Alltech Corp., Deerfield, IL, USA). A 50/30 DVB-CAR-PDMS fiber (Supelco, Bellofonte, PA) was inserted into the vial and let to adsorb volatiles for 30 min at 40°C and then transferred to the injector port (250°C) where desorption occurred in 2 min. *Splitless* mode was selected as injection mode. GC-MS analyses were performed on a GC 6890 (Agilent Technologies, Palo Alto, CA) coupled to an Agilent MSD 5973 Network detector using a HP-INNOWAX capillary column (60 m × 0.25 mm, 0.25 μm, J&W Scientific Inc., Folsom, CA, USA) as reported by Tufariello et al. (2019). Concentrations of volatiles were assessed by the internal standard method. The GC/MS data subjected to subsequent statistical processing consist of the average values of three replicates obtained by carrying out three different extraction procedures for each sample.

### 2.6. Sensory analysis

A trained panel of ten judges between 40 and 65 years of age, experienced in alcoholic beverage sensory evaluation, carried out a Quantitative Descriptive Analysis (QDA) in a room free of noise, odors and with white light. Fifty milliliters of each beer were served to the panelists in crystal goblets at a temperature of 5 ± 1°C. The parameters evaluated by the judges were selected among those found in the literature (Baiano et al., 2023) and those generated by the panel to both give a complete product description and avoid overlapping. Data were collected using a combined profile sheet including 3 appearance (color, amount, and persistence of foam), 12 olfactory (malty, hoppy, floral, fruity, spicy, honey, caramel, yeast, smoked, aromatic herbs; olfactory finesse and the overall olfactory intensity), 11 gustatory (sweetness, bitterness, saltiness, acidity/sourness, malty, hoppy, floral, fruity, spicy, toasted, alcoholic), and 2 tactile (effervescence and body) parameters. Panelists were also asked to evaluate the overall quality of each beer. All descriptors and the overall quality were evaluated on a 5-point scale with the exception of those referred to foam color, which were evaluated on a 4-point scale (Supplementary Table 1). Drinking water was used for mouth-rinsing between tastings.

### 2.7. Consumer test

The consumer test was conducted with 42 consumers (female 29 and men 13), with age ranging between 21 and 75 years. The prerequisites for participating in the study were that the individual habitually consumed beer and they were selected on the basis of other criteria, such as not reporting any conditions affecting the senses of sight, taste or smell, The test was conducted in the sensory laboratory from the School of Agricultural, Forestry, Food and Environmental Sciences of Basilicata University using individual sensory booth equipped with sensory data collection software (Smart Sensory box version 2.3.5., Smart Sensory Solution, Italy). The test was conducted to assess liking for appearance, odor, taste, and overall liking, using a using a 9-point hedonic scale in which 1 = dislike extremely, 5 = neither like nor dislike and 9 = like extremely (Peryam and Pilgrim, 1957). The samples were randomly coded with 3-digit numbers, and the order of the sample presentation was randomized to minimize first-serving order bias. Each participant received about 15 mL of sample, served at refrigerator temperature (4°C); water and crackers were used as palate cleansers.

### 2.8. Statistical analysis

One-way ANalysis Of VAriance (ANOVA) applied to chemical, sensory, and volatolomic data determined significant quantitative differences among samples. Differences were considered statistically significant for p-values of the null hypothesis less than or equal to 0.05. Partial Least Squares Correlation (PLSC) was applied to volatile compound data and sensory analysis data to study their multivariate covariance. PLSC (Tucker, 1958; Streissguth et al., 1993) is a multivariate statistical method used to analyze, through correlation, the relationship between two sets of variables. Volatolomic data were also used to create a clustergram consisting of a heatmap and dendrograms. The heatmap indicated, in a scale of false colors, the standardized concentration of each volatile compound for each of the samples considered. Samples and volatiles were organized according to the corresponding hierarchical clustering dendrograms. Euclidean distance as metric and unweighted average distance as linkage criterion, were considered. The calculations and visualization of the ANOVA, PCA, PLSC, and clustergrams results were performed using MATLAB Version: 9.14.0 (R2023a).

## 3. Results and discussion

### 3.1. Starter evolution during fermentation

The strains growth was monitored during the fermentation process by a microbiological assay (Table 1). The viable count on WL medium, followed by ITS-RFLP analysis, revealed that all the isolated colonies during the process belong to *S. cerevisiae* species in order to confirm that no contamination occurred during the fermentation trials. The theoretical inoculum ratio resulted as planned; in fact, 24 h after inoculation, for all the trials the yeast population ranging between 7.1 and 7.9 Log CFU/mL. At this time, the three indigenous strains reached levels higher than those of the control strain US-05 (7.15 Log CFU/mL); the highest count (7.94 Log CFU/mL) were displayed by the strain CHE-3. At the end of primary fermentation, an increase of yeast population was observed for P4 and CHE-3 strains (0.8 and 0.2 Log cycles, respectively), a slight decrease to 6.96 Log CFU/mL was observed for the control strain US-05, whereas a 0.7 Log cycles decrease was registered for the strain TA4-10. After the secondary fermentation, the end of monitoring, all the fermentations showed a decrease of yeast levels; the highest value (7.05 Log CFU/mL) was detected for CHE-3 strain, whereas the lowest level was found for the control strain US-05, which was the strain showing the lowest count along all the process. However, the high yeast vitality found during the process for all the starters is crucial for improving fermentation and product quality as it is related to the efficiency and predictability of fermentation. This aspect, together the presence of only *S. cerevisiae* cells, are pivotal for beer quality, in particular for craft beers, which are more prone to spoilage than beer produced in large-scale breweries, in consequence of absence of pasteurization or sterile-filtration processes.

**TABLE 1:** Growth of inoculated starters, expressed as Log UFC/mL, during the different steps of IGA production.

	TA4-10	P4	CHE-3	US-05
After 24 h	7.71 ± 0.71	7.72 ± 0.57	7.94 ± 0.14	7.15 ± 0.35
End primary fermentation	7.02 ± 0.35	8.51 ± 0.58	8.15 ± 0.25	6.96 ± 0.43
After refermentation	6.18 ± 0.30	6.57 ± 0.26	7.05 ± 0.64	5.59 ± 0.35

### 3.2. Physicochemical analyses

The IGA beers were analyzed for the main quality attributes, such as ethanol content, attributes related to turbidity, color and bitterness (Table 2). The ethanol ranged between 4.4 and 5.1 (% v/v), with the highest content in beer fermented with the commercial starter, this result being likely correlated with the very low maltotriose residual found in this beer.

**TABLE 2:** Principal quality parameters of the IGA beers obtained in the micro brewing plant.

Sample	Final gravity	Ethanol (%v/v)	Bitterness (IBU)	Color (EBC-unit)	Turbidity (NTU)
CHE-3	1011.13 ± 0.18^a^	4.88 ± 0.61^a^	12.60 ± 0.32^a^	18.37 ± 0.31^a^	15.63 ± 0.25^c^
P4	1010.12 ± 0.18^b^	4.44 ± 0.16^ab^	13.4 ± 1.1^a^	16.49 ± 0.16^b^	34.23 ± 0.12^a^
TA4-10	1009.25 ± 0.35^b^	4.76 ± 0.03^a^	9.19 ± 0.37^b^	16.46 ± 0.06^b^	33.7 ± 1.2^a^
US-05	1009.93 ± 0.11^b^	5.11 ± 0.20^ac^	8.14 ± 0.16^b^	20.73 ± 0.11^c^	54.53 ± 0.12^b^

Data are reported as mean ± standard deviation of two independent replicates. Different superscript letters indicate significant differences (*p* < 0.05) among the four samples.

All the beers presented a bitterness (International Bitterness Units, IBU) ranging between 8 and 13.3. The lowest values, found in TA4-10 and US-05 samples (9 and 8, respectively), were slightly lower than indications given by the Beer Judges Certification Program (BJCP), whereas IBU values significantly higher were found in beers fermented with CHE-3 and P4. Hops are the raw material in beer mainly responsible for beer bitterness. Indeed, it provides α-acids, which during the technological process are transformed into the more bitter iso-α-acids. By considering that the variety and quantity of hop were kept constant among all beer samples, the difference in beer IBU might be correlated to the different capacity of yeast strain used for fermentation to preserve the α-acids from hop. These results enhance the role of starter on beer bitterness, conversely to data reported in other studies in which no differences in IBU were found among beers fermented with different starters (Matukas et al., 2022). Beer color, a commercial parameter crucial for quality product, is affected by amount and quality of the used malt; it usually can range from pale (0–15 EBC), amber (15–35 EBC), and dark (35–80 EBC). The analyzed samples had a color in the range 15-35 EBC; the highest value was found in beer produced with the commercial starter, whereas the lowest levels were found in P4 and TA4-10 beers, which were very similar among them. A similar trend was observed also for turbidity levels, with similar values for IGA produced by inoculating P4 and TA4-10 strains, and the highest turbidity (54.53 NTU) in US-05 beer. The turbidity is another parameter important for beer quality and for consumer acceptability. Following the EBC (European Brewery Convention) indications, beer is considered very hazy when it shows a turbidity level higher than 32 NTU (De Francesco et al., 2021); the IGAs produced with P4 and TA4-10 strains showed values slightly higher than this level, whereas very low turbidity was observed in CHE-3 beer.

The profile of carbohydrate present in the produced beers are shown in Table 3A. The monosaccharides maltose and glucose were almost very fermented. In fact, no residual maltose and glucose were found in all the produced beers indicating that the four strains efficiently utilized maltose during fermentation. Otherwise, some fructose residual was found in all the produced IGA beers. Furthermore, the three crossover strains did not proficiently catabolize the maltotriose, whereas a low residue of this compound was found in beer produced with reference strain US-05. The catabolism of maltotriose in yeasts is species-specific (Li et al., 2020). However, most yeast species catabolize maltotriose slowly and often incompletely (Cubillos et al., 2019). The beers contained a maltodextrin level very similar to wort, as expected, by considering that *S. cerevisiae* yeast is not able to utilize the dextrins (Armijo et al., 2016), with exception of *S. cerevisiae* var. *diastaticus*. As reported in Table 3B, the highest glycerol content was found in P4 sample, while acetic acid was higher in sample fermented with TA4-10 (isolated from grape must) and US-05 strains. The glycerol concentration in beer is affected, among other factors, by the yeast strain used and condition of the fermentation process. A higher glycerol concentration affects the sensory characteristics of the product as it enhances the perception of a sweet taste and increases product viscosity (Zhao et al., 2015). Citric, malic and tartaric acids derive from wort and/or from added grape must and all the fermentation samples showed not statistically different amounts of these organic acids, except for tartaric acid.

**TABLE 3A:** Carbohydrate content of experimental beers (g/L).

Samples	Maltodextrins	Maltotriose	Maltose	Glucose	Fructose
Wort	15.14 ± 0.12	15.17 ± 0.14	34.56 ± 0.25	17.13 ± 0.14	13.16 ± 0.11
TA4-10	14.54 ± 0.06	14.58 ± 0.19	ND	ND	0.49 ± 0.14
CHE3	14.53 ± 0.03	13.1 ± 1.2	ND	ND	0.35 ± 0.04
P4	14.6 ± 1.0	13.53 ± 0.83	ND	ND	0.33 ± 0.04
US-O5	15.32 ± 0.26	5.33 ± 0.16*	ND*	ND	0.35 ± 0.04

Data are reported as mean ± standard deviation of two independent replicates; ND, not detected; *p < 0.05.

**TABLE 3B:** Organic acids, glycerol and ethanol of experimental beers (g/L).

Samples	Citric acid	Malic acid	Tartaric acid	Succinic acid	Lactic acid	Acetic acid	Glycerol
Wort	1.81 ± 0.11	2.27 ± 0.09	0.82 ± 0.10	ND	0.05 ± 0.01	0.38 ± 0.01	0.44 ± 0.03
TA4-10	2.09 ± 0.04	1.86 ± 0.02	0.48 ± 0.00	0.76 ± 0.19	0.36 ± 0.03	0.24 ± 0.08	4.78 ± 0.06
CHE3	2.01 ± 0.17	2.56 ± 0.14	0.50 ± 0.16	0.75 ± 0.05	0.44 ± 0.03	0.14 ± 0.03	4.26 ± 0.39
P4	1.27 ± 0.18	2.26 ± 0.04	0.64 ± 0.06	1.16 ± 0.00	0.32 ± 0.01	0.19 ± 0.02	5.50 ± 0.05
US-O5	2.08 ± 0.59	2.04 ± 0.81	0.68 ± 0.23	0.78 ± 0.04	0.34 ± 0.02	0.25 ± 0.03	4.40 ± 0.23

Data are reported as mean ± standard deviation of two independent replicates; ND, not detected.

### 3.3. Volatile profile of IGA beers

Thirty-one volatile compounds grouped in the esters, alcohols, terpenes and volatile acids families, were identified in the analyzed IGA beers (Table 4). The alcohols group was quantitatively the most important one, with a total concentration ranging from 332.03 mg/L in CHE-3 to 411.54 mg/L in P4. Among alcohols, significant differences (*p* < 0.05, *p* < 0.01) were found for 2-methyl-1-butanol, 3-methyl-1-butanol and isobutanol. The higher alcohols may influence the sensory profile of beer by giving an alcoholic or solvent-like aroma and a warm mouthfeel (Meilgaard and Peppard, 1986). These compounds play a direct role in taste perception or through interactions with other beer components due to the synergistic and antagonistic effects that these compounds induce on taste perception. Among the identified compounds belonging to this class, only the 3-methylbutanol (solvent and fruity flavor) was above the perception threshold, as previously reported in a study aimed to a characterization of a wide number of commercial IGA samples (De Francesco et al., 2021). This compound influences the drinkability of beer since sensory analysis describes beer flavor as heavier when the content of amyl alcohol increases (Olaniran et al., 2017). Furthermore, 2-methylbutanol and 3-methylbutanol were found at level significantly higher in beers obtained with indigenous strains than beer fermented with the reference strain US-05. These alcohols are produced in fermented beverages as a result of the metabolism of amino acids, such as isoleucine and valine. Differences found in their concentrations despite the use of wort with identical composition as fermentation medium, which should have the same concentration of the above-mentioned amino acids, may indicate that indigenous strains are characterized by a different metabolism compared to typical brewer yeast US-05.

**TABLE 4:** Organic volatile compounds determined in IGA beers by means GC-MS and GC-FID.

Volatile molecules (mg/L)	US05	sd	TA4_10	sd	P4	sd	CHE-3	sd	OTH (mg/L)	Sensory notes	One-way Anova
**Esters**											
Ethyl acetate	0.07	0.01	0.05	0.02	0.08	0.02	0.06	0.02	25–30	Fruity-solvent-apple	ns
Isoamyl acetate	0.08	0.02	0.27	0.04	**0.65**	0.14	0.21	0.08	0.6–2	Banana	*
Ethyl hexanoate	**0.20**	0.04	**0.46**	0.02	**1.29**	0.34	nd		0.2–0.23	Apple-fruit	*
Hexyl acetate	nd		0.06	0.02	0.06	0.02	nd			Fruit	*
Methyl octanoate	0.05	0.01	0.02	0.01	0.02	0.00	0.02	0.01			ns
Ethyl octanoate	0.50	0.17	nd		0.38	0.07	nd		0.9–1.0	Apple-aniseed-cheesy	*
Methyl decanoate	0.06	0.02	0.06	0.02	0.07	0.03	0.05	0.02			ns
Ethyl decanoate	0.06	0.02	0.08	0.03	0.32	0.01	0.08	0.01	1.5	Apple	**
Ethyl-9-decenoate	0.04	0.02	0.07	0.02	0.09	0.03	0.05	0.02			ns
Methyl dodecanoate	0.11	0.03	0.12	0.03	0.09	0.03	0.10	0.02			ns
Phenylethyl acetate	0.03	0.01	**0.29**	0.06	**0.30**	0.01	0.18	0.04	0.25	Roses-honey	*
Ethyl dodecanoate	0.06	0.01	0.06	0.01	0.06	0.02	0.05	0.02			ns
Sum	1.26		1.54		3.41		0.80				
**Alcohols**											
n-propanol^	15.5	3.4	11.49	0.86	14.39	0.98	11.43	0.62			ns
Isobutanol^	90.1	4.1	91.3	5.6	94.5	4.8	61.8	4.1	100	Solvent	*
n-butanolo^	122	13	123.2	8.7	142.8	5.4	115	11			ns
2 methyl butanol^	29.9	2.5	40.3	2.9	53.4	5.2	37.9	3.8	65		*
3 methyl butanol^	**90.4**	7.2	**133.1**	8.9	**119.9**	11.5	**117.1**	9.8	70	Alcoholic-banana	*
1-hexanol	nd		nd		0.05	0.01	0.03	0.01	8	Green	ns
Phenylethanol	0.28	0.08	0.40	0.02	0.94	0.25	0.38	0.11	40-100	Roses	ns
2-ethyl-1-hexanol	0.05	0.01	0.06	0.02	0.04	0.01	0.06	0.02	8	Mild green	ns
Sum	332.72		388.32		411.58		332.05				
**Volatile acids**											
Hexanoic acid	0.03	0.01	0.03	0.01	0.08	0.02	0.03	0.01	5		ns
Octanoic acid	0.20	0.07	0.27	0.01	0.45	0.17	0.20	0.06	10		ns
n-decanoic acid	0.01	0.00	0.08	0.03	0.11	0.01	0.04	0.01	10		ns
Linoleic acid	0.07	0.01	0.03	0.01	0.03	0.01	0.05	0.02			
Sum	0.31		0.41		0.67		0.32				
**Terpenes**											
Linalool	**0.03**	0.01	**0.02**	0.01	**0.02**	0.01	**0.04**	0.01	0.001-0.1	Green-lemon	ns
Citronellol	**0.07**	0.01	**0.04**	0.02	**0.05**	0.02	**0.04**	0.01	0.009-0.04	Rose-sweet	ns
Nerolidol	0.06	0.01	0.04	0.01	nd		nd		1	Rose-apple-citrus	ns
Sum	0.16		0.10		0.07		0.08				
**Other compound**											
Acetoin^	3.35	0.46	12.94	2.47	nd		10.83	0.68	150		*
Acetaldehyde^	30.7	2.2	31.4	3.8	36.9	4.1	34.1	2.8			
Styrene	0.05	0.02	nd		0.04	0.02	0.05	0.01			ns
2-octanone	0.04	0.01	0.03	0.01	0.03	0.01	0.04	0.02			ns
Sum	34.17		44.36		36.89		45.00				

sd, standard deviation; nd, not detected; OTH, odor threshold; ^molecules determined by GC-FID; *significant differences *p* ≤ 0.05, **significant differences *p* ≤ 0.01. In bold the concentrations above odor threshold.

The higher alcohols are also involved in the biochemical pathways leading to the synthesis of esters, which is another important group for beer aroma. The most described flavor-active esters in beer are ethyl acetate (solvent-buttery like aroma), ethyl caproate, ethyl caprylate (sour apple-like flavor and aroma), isoamyl acetate (fruity, banana aroma), isobutyl acetate, phenylethyl acetate, and ethyl octanoate (honey, fruity, roses, flowery aroma). In our samples, ethyl esters and acetates represented the second most abundant group of volatile compounds (Table 4), ranging from 0.72 mg/L in CHE-3 to 3.28 mg/L in P4. Significant differences in concentrations of different flavor-active esters, such as isoamyl acetate, ethyl hexanoate, hexyl acetate, ethyl octanoate, ethyl decanoate and phenylethyl acetate were found among beers obtained by different starters. Indeed, ester concentration is the result of the enzymatic balance of synthesis by alcohol acetyltransferases (AATases) and acyl-CoA/ethanol O-acyltransferases (AEATases) and breakdown by esterases. The activity of these enzymes is strain-specific (Marconi et al., 2016). The ethyl octanoate concentration was the highest in the US-05 sample, whereas the P4 beer showed the highest amount of the other above esters, probably due to the fermentative performance of this yeast strain and/or the higher ester synthase activity than esterase activity of P4 strain.

Phenyl acetate and ethyl hexanoate were above their perception thresholds for TA4-10 and P4 beers, contributing with rose, honey, and green notes to the aroma of these samples. Among esters, ethyl hexanoate, isoamyl acetate and phenyl acetate were in concentrations above their respective perception thresholds in P4 contributing significantly to the overall aroma (Rossi et al., 2014). However, both sensory and gas-chromatographic analyses revealed that esters might affect beer flavor also if these aromatic compounds are present at concentration below their individual threshold values (Rodrigues et al., 2008). It was reported that the presence of the different esters could play synergistic effects on individual flavors, interfering with the overall aromatic profile of the beer. Furthermore, since most esters are present in concentrations ranging in the threshold value, little variations in their concentration may significantly affect the organoleptic properties of the beer (Humia et al., 2019).

Acids are the third most abundant group in beers studied, but not statistically significant differences have been detected in the amount found in the different samples. The acids are produced during fermentation process but their amount depend both on yeast activity and on the composition of raw materials (Schreier and Jennings, 1979).

Three terpenes have been found in IGA beers, i.e., linalool, citronellol and nerolidol. In all samples, linalool and citronellol were found in concentrations above their odor thresholds, thus enhancing the aromatic profiles of IGA beers with fruity and floral notes. Moreover, IGA are beer made with a variable percentage of grape must, which is mainly characterized by primary aromatic compounds derived from grapes, such as terpenes, sulfur compounds, pyrazines, and norisoprenoids (Crupi et al., 2010; Slegers et al., 2015).

**FIGURE 1:**
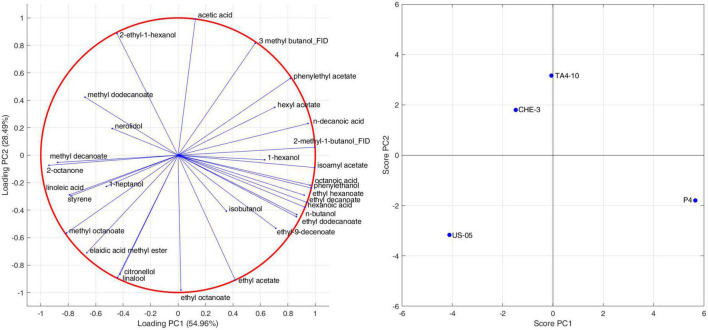
Pearson principal component analysis performed on GC-MS data for the detection of volatile compounds.

**FIGURE 2:**
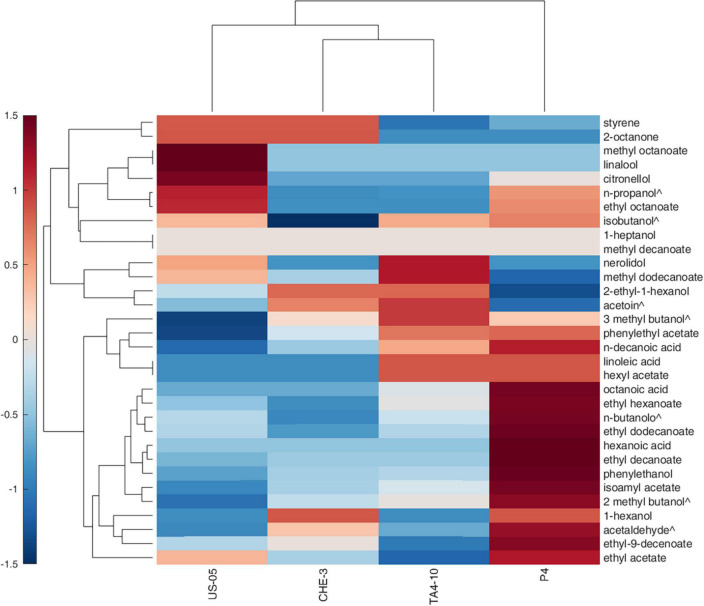
Hierarchical clustering heat map performed on the GC-MS normalized data (distance measure using Euclidean and clustering algorithm using average). Each colored cell on the map corresponds to a concentration value in the data table, with samples in columns and metabolites in rows. Molecules indicated with ^∧^ are detected by GC-FID.

To highlight the effects of yeast strains on volatile molecules concentration, a *Pearson* principal component analysis (PCA) was carried out. Figure 1 shows the projection of the beer samples on a factor plane in which the first two principal components explained 54.96 and 28.49% of the total variance, respectively. US-05 is described by negative PC1 and PC2, and it was associated with higher values of methyl octanoate and decanoate, 2 octanone, 1-heptanol, linalool and citronellol. P4 is characterized by positive PC1 and negative PC2 due to higher concentrations of most volatiles identified. Indeed, it was the sample with the most complex and rich volatile profile. Finally, TA4-10 and CHE-3 group together and were characterized by a positive value of PC2 that is due to a greater content in acetic acid, 2-ethyl-1-hexanol and nerolidol. Overall, our results, based on the quantification of volatile compounds, showed that the yeast strain has a statistically significant impact on the majority of target volatile compounds. This result is in line with previous research showing that the yeast strain is a significant factor in the formation of main volatile compounds in beer and starter selection is a key factor to modulate beer characteristics (De Simone et al., 2021; Matukas et al., 2022). A clustergram, which consists of a heatmap of standardized compound concentrations and dendrograms, was utilized as a tool for exploratory multivariate analysis of the volatile profiles of beers. This clustergram provided valuable insights into the relationships between the different yeast strains tested and the quantified compounds (as shown in Figure 2). The dendrograms, both by samples and by compounds, were generated through a hierarchical clustering analysis using the Euclidean metric to assess similarity/dissimilarity and the unweighted average distance as the linkage criterion. As shown in Figure 2, the red color in the heatmap indicated the highest standardized concentrations of each substance while blue represents the lowest one. The beers produced with the four yeast strains constitute four independent clusters, they being dissimilar to each other’s. Consistent with the PCA results, however, samples CHE-3 and TA4-10 are more similar to each other.

Overall, the samples were characterized by different standardized concentrations of the analyzed volatiles. Indeed, US-05, the commercial strain used as control, was characterized by higher production of molecules ranging from styrene to isobutanol; sample obtained with P4 strain showed high values of compounds ranging from phenylethyl acetate and ethyl acetate; however, this beer was characterized by the highest amounts of most detected volatiles, confirming the interesting findings of this strains previously obtained during brewing trials at laboratory scale (Capece et al., 2018, 2021). The TA4-10 sample is distinguished from the others by higher concentrations of volatiles ranged from nerolidol and hexyl acetate.

Finally, CHE-3 beer appeared to be the sample with fewer dominant volatile molecules, including 2-octanone, styrene, isoamyl alcohols, 2-ethyl-1-hexanol, acetoin and 1-hexanol. Our results underscore the ability of the yeast strain to influence the volatolomic profile, from a quantitative point of view, by affecting the sensory perception of notes associated with the detected molecules.

### 3.4. Descriptive sensory analysis

In order to investigate the impact of the three cross-over strains on the sensory quality of beers obtained by using malt fortified with grape must, a sensory analysis was carried out. This study offers valuable insight into the sensory response of Italian consumers on this new version of beer, which has a particular aromatic complexity strongly influenced by the combination of malt and grape must.

**FIGURE 3:**
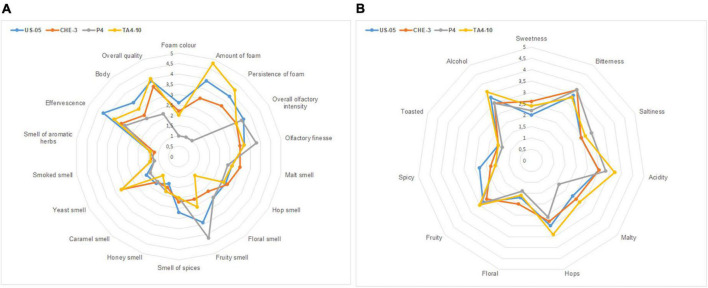
Radar plots of visive, tactile, olfactory **(A)** and gustative **(B)** descriptors indicated by panelists.

The Supplementary Table 2 reported the mean values of sensory descriptors used by trained panelists to describe visive, tactile, olfactory and gustative aspects of beers. Sensory analysis revealed that the yeast strain can significantly (*p* < 0.05) affect some of the attributes considered, including foam color, amount and persistence of foam, effervescence, olfactory finesse, floral, yeast and fruity smell, acidity, and finally overall quality. Spider plots for the aroma (visive, tactile, olfactory attributes) and taste profiles, reporting the mean values of samples, are shown in Figures 3A, B.

The comparison of the ANOVA results (Supplementary Table 2) and the sensory radar plots (A-B) (Figure 3), indicated that all IGA beers showed high scores (≥3) for “olfactory finesse” and “olfactory intensity,” probably due to the complexity of volatile profiles characterized by molecules belonging to different classes. Some of them, having an OAV > 1, directly affected odor perception, while the molecules having an OAV < 1 might have contributed to the beer flavor through the additive effects of compounds with similar structure or odor (Francis and Newton, 2005). All beers were characterized by medium-high scores for fruity and floral descriptors, which are influenced by wine must addition and then by yeast action (De Francesco et al., 2021). The floral notes are associated with terpenes, varietal molecules found in grapes, while the fruity character is mainly related to alcohols (i.e., phenyl ethanol) and esters, fermentation by-products. P4 beer was judged to have the higher score for fruity (4.25) than others, probably linked to the high ester content. Yeast smell was a descriptor with the higher score (3.25) in CHE-3 and TA4-10. Figure 3B illustrated the radar plot of taste profile. Among gustative descriptors, acidity was perceived in scores significantly different, with high values (3.75) in TA4-10. Medium scores for sweetness are detected in all IGAs, ranged from 2 (US-05) to 2.5 (TA4-10; CHE-3), probably due to low sugar residual content.

**FIGURE 4:**
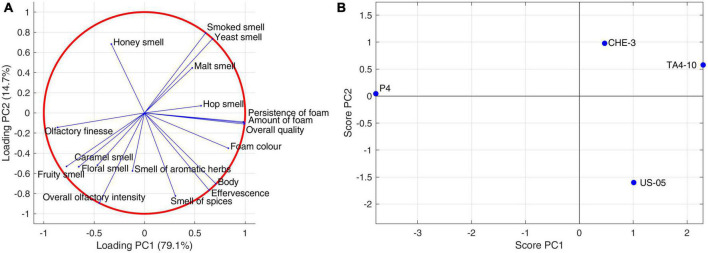
Principal component analysis performed on sensory (olfactive-tactile and visive) descriptors: **(A)** sensory data correlation circle; **(B)** sensory data score plot.

A PCA was applied to analyze the covariance of sensory descriptors judged by panelists (Figures 4A, B). The first two principal components explained the 94.08% of the variance (78.74 and 15.34% for PC1 and PC2, respectively). PC1 components separated P4 from the other samples. Indeed, P4 beer was positioned at negative values of PC1, which can be related to its olfactory finesse and overall olfactory intensity, as well as caramel, floral and fruity descriptors intensity. By contrast, CHE-3 and T4-10, clustering in the same quadrant delimited by positive PC1 and PC2, contributed to variance thanks to smoked, yeast, malt and hop olfactory descriptors. The body, effervescence, spices smell and then particular characteristics of foam, described by positive PC1 and negative PC2, influenced US05. Undoubtedly, foam is one of the key attributes of beer that differentiates it from other beverages. Different factors, endogenous or exogenous, significantly affect foam consistency and persistence, including protein components, hop acids, non-starch polysaccharides, metal ions, lipids, proteolytic enzymes, ethanol concentration, basic amino acids, as well as malting and brewing processes (Blasco et al., 2011; Donadini et al., 2011). Moreover, yeast proteins also play a secondary role in enhancing the foam (Lao et al., 1999). In particular, the beer produced with the TA4-10 strain was characterized by the visive attributes “amount and persistence of foam,’ the gustative descriptor “acidity,” and then “overall quality,” whereas the P4 one was distinguished by olfactory notes “fruity smell and floral” and “olfactory finesse.”

**FIGURE 5:**
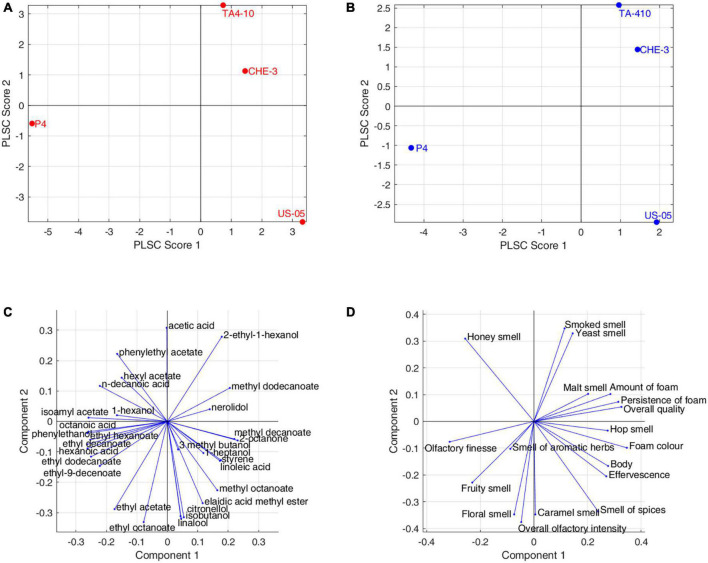
Partial least squares correlation between volatolomic and olfactory profiles: **(A)** volatolomic score plot; **(B)** olfactory score plot; **(C)** volatolomic loadings plot; **(D)** olfactory loadings plot.

In our research, Partial Least Square-Correlation (PLSC) analysis was applied to study the relationship between two different groups of data obtained with different techniques, as reported in literature (Aznar et al., 2003; Vilanova et al., 2010; Delgado et al., 2022; Palombi et al., 2023). In particular, PLSC was used to study the correlation between volatile compounds and olfactive and visive descriptors scores and Figure 5 showed the score and loading plot of the first two PLSC components for the volatiles/olfactive-visive descriptors. The first two components explained, respectively the 48.9% and 32.5% of the multivariate covariance between the two datasets. In detail, the correlation coefficient between the scores of the first component of volatiles data and the scores of the first component of sensory data was 0.9873 (*p*-value = 0.013), whereas for the second components the correlation coefficient was 0.9825 (*p*-value = 0.0175).

The P4 strain contributed to the overall correlation between the datasets with higher concentration of ethyl esters (ethyl hexanoate, ethyl decanoate, ethyl dodecanoate), acetate esters (isoamyl acetate, ethyl-9-decenoate), hexanoic acid. These molecules were correlated to the fruity smell and olfactory finesse, indicating the contribution of esters on fruity notes in IGA beers. US-05 was positively correlated with terpenes (citronellol, linalool), ethyl octanoate, ethyl acetate, isobutanol and was characterized by “floral, spicy and caramel” notes and more pronounced “overall olfactory intensity.”

Finally, CHE-3 and TA4-10, differed from the others for a positive correlation with few volatiles, including 2-ethyl-1-hexanol, acetic acid, methyl dodecanoate and nerolidol, while, on the sensorial-olfactory level, they displayed high correlation with “smoked, yeast and malt smell” and then a good quality of foam.

### 3.5. Consumer test

In the last step of the research, the beer samples were submitted to evaluation of consumer liking. This is fundamental to identify the reason for the success (or failure) of a product and its market opportunities, giving useful indication about the suitability of a testing beer for full scale production in the breweries. The panel was composed of 69% females and 31% males, with an average consumer age of 44 ± 14 years old and ranged from 21 to 75 years old. The recruited consumers rated the following parameters: appearance liking, taste liking, odor liking and then overall liking. The mean values and standard deviations for each descriptor are reported in Supplementary Table 3. One-way ANOVA and multi-way ANOVA (with and without factors interaction) were conducted to explore the hedonic responses of the consumers. The factors taken into account were the different beer samples, the gender and the consumer age (this divided in three classes: 18 to 31 years, 31 to 45 years and 46 to 60 years). Results showed no significant differences (*p* < 0.05), for beer sample, gender and age class. No statistically significant interactions were found.

**FIGURE 6:**
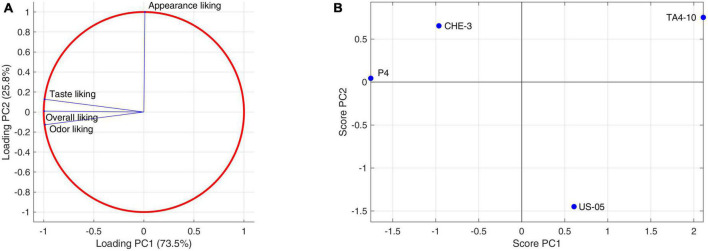
Principal component analysis performed on consumers descriptors: **(A)** consumers liking data correlation circle; **(B)** consumers liking data score plot.

A PCA was applied also to liking attributes judged by the consumers (Figures 6A, B). Although the samples differences are not statistically significant, the plane identified by the first two components, describing 99% of the total variance, shows a clear differentiation of the samples. PC1 (73.5%) is closely related to smell, taste, and overall liking, attributes that are highly correlated with each other. Sample scores allow us to establish a higher liking for P4 and CHE-3 beers as regards these attributes, with above-average liking values. Lower approval ratings were associated with US05 and TA4-10 beers. PC2 (25.8%), on the other hand, is almost exclusively linked to appearance, an attribute perceived to a greater extent in the CHE-3 and TA4-10 samples, to an average extent in P4, and to a lesser extent in the control US-05.

Both sensory analysis and consumers’ test indicated that beer obtained by using P4 (Figures 4B, 6B, respectively), characterized by higher fruity and floral notes, which significantly influenced the finesse and olfactory intensity, was the most appreciated beer for smell, taste, and overall quality. On the other hand, the CHE-3 and TA4-10 samples, with better visual properties related to the consistency and color of the foam influencing the overall quality, were appreciated above all for their appearance. Given all that, the cross-over strains tested contributed positively to the improvement of the various sensory aspects of the beers produced, validating the results previously obtained during laboratory-scale fermentations (Siesto et al., 2023).

## 4. Conclusion

In the present study, the applicability of *S. cerevisiae* strains selected from different food matrices for the production of craft IGA beers was investigated for the first time through a multidisciplinary chemistry-multivariate approach. Overall, the strains tested showed the ability to degrade maltose during fermentation and contribute to the qualitative complexity of the final products. In particular, beer obtained by inoculating P4 strain showed high content of some classes of volatile molecules, including alcohols, esters, and volatile acids, as shown by the cluster heatmap and PCA. In agreement with the chemical data, the sensory analysis performed by a trained panel on P4 sample revealed more pronounced fruity and floral notes. On the other hand, TA4-10 differed from the control (US-05) and from the other samples (CHE-3, P4) especially for the visual attributes “quantity and persistence of foam,” for the taste descriptor “acidity” and for its “overall quality.”

In terms of consumer acceptability, the overall liking, odor-taste-appearance liking were not significantly different among four beers, but consumer group showed a preference for P4, concerning odor-taste-overall liking, whereas CHE-3 and TA4-10 were preferred for appearance. An interesting result is the highest consumer preference toward beers obtained by using indigenous *S. cerevisiae* strains.

Moreover, the PLSC clearly indicated how crucial the interaction between analytical and sensory data is in obtaining a snapshot of the overall quality of the fermented beverage, which is useful in standardizing a fermentation process. In conclusion, this work represents the first phase of a wider project for the qualitative improvement of IGA beers, which will industrially employ indigenous cross over starter cultures as potential approach to improve the organoleptic profile of this innovative beverage and to tie it to the culture and history of the production area.

## Data availability statement

The original contributions presented in this study are included in the article/Supplementary material, further inquiries can be directed to the corresponding authors.

## Author contributions

MT: conceptualization, investigation, data curation, writing–original draft, and writing–review and editing. LP: data curation, formal analysis, data acquisition, software, validation, writing–original draft, and writing–review and editing. FG: conceptualization, writing–original draft, and writing–review and editing. CG, ABr, and FGe: data acquisition and writing–review and editing. AC: conceptualization, funding acquisition, investigation, data acquisition, writing–original draft, and writing–review and editing. GS and RP: investigation, methodology, data acquisition, data curation, and writing–review and editing. ABa: data acquisition, writing–original draft, and writing–review and editing. All authors contributed to the article and approved the submitted version.
