# Supplementary material for: A multivariate approach to explore the volatolomic and sensory profiles of craft Italian Grape Ale beers produced with novel Saccharomyces cerevisiae strains
Source: Front Microbiol. 2023 Jul 27;14:1234884. doi: 10.3389/fmicb.2023.1234884 (PMC10414987; doi:10.3389/fmicb.2023.1234884)
Supplement: Supplementary file 1 [file Data_Sheet_1.PDF]

Supplementary Material

A multivariate approach to explore the volatolomic and sensory profiles of craft Italian Grape Ale beers produced with novel *Saccharomyces cerevisiae* strains

Rocchina Pietrafesa[1†], Gabriella Siesto[1,2†], Maria Tufariello[3*], Lorenzo Palombi[4*], Antonietta Baiano[5], Carmela Gerardi[3], Ada Braghieri[1], Francesco Genovese[1], Francesco Grieco[3] and Angela Capece[1,2]

*** These authors contributed equally to this work and share correspondence:**
Maria Tufariello, maria.tufariello@ispa.cnr.it
Lorenzo Palombi, l.palombi@ifac.cnr.it

† **Equal contribution and first authorship:** These authors contributed equally to this work and share first authorship

Table S1: Evaluation of sensory attributes

Sensory attributes	Description	Evaluation	Scores
Foam colour	Colour intensity and hue	White	1
		Rose	2
		Cream	3
		Capuchin	4
Effervescence	Number of bubble chains	Absent	1
		Poor	2
		Medium	3
		Good	4
		Abundant	5
Foam amount	Amount of foam immediately after pouring	Absent	1
		Poor	2
		Medium	3
		Good	4
		Abundant	5
Foam persistence	The time needed for disappearance of the initial foam	Evanescent	1
		Little persistent	2
		Moderately persistent	3
		Persistent	4
		Extremely persistent	5
Specific olfactory descriptors/Overall olfactory intensity	Strength of the specific olfactory/overall olfactory sensation	Absent/Very weak	1
		Weak	2
		Medium	3
		Strong	4
		Very strong	5
Olfactory finesse	Harmonious, well-balanced combination of all olfactory elements	Very bad	1
		Bad	2
		Good	3
		Very good	4
		Excellent	5
Specific gustatory descriptors	Strength of the specific gustatory sensation	Absent/Very weak	1
		Weak	2
		Medium	3
		Strong	4
		Very strong	5
Body	It is defined by the weight, texture, and mouthfeel of the cider	Skinny	1
		Weak	2
		Light-bodied	3
		Medium-bodied	4
		Full-bodied	5
Overall quality	The overall impression of the product deriving from the evaluation of all the sensory attributes	Very bad	1
		Bad	2
		Good	3
		Very good	4
		Excellent	5

Table S2: Sensory descriptors indicated by trained panelists

	Sensory Descriptors	**US05**	**±*sd***	**CHE-3**	**±*sd***	**P4**	**±*sd***	**TA410**	**±*sd***
Visual descriptors	Foam color	**2.5b**	*0.84*	**2ab**	*1.03*	**1a**	*0.02*	**2ab**	*0.00*
	Amount of foam	**3.75bc**	*0.88*	**3b**	*0.67*	**1a**	*0.02*	**4.75c**	*0.42*
	Persistence of foam	**3.75bc**	*0.42*	**3.25b**	*0.42*	**1a**	*0.02*	**4.25c**	*0.42*
Tactile descriptors	Effervescence	**4.25b**	0.42	**3.25a**	0.42	**3a**	0.03	**3.5a**	0.52
	Body	3.25a	0.84	2.5a	0.52	2.5a	0.52	3a	0.04
Olfactory Intensity	Overall olfactory intensity	3.5a	0.52	3.25a	0.42	3.25a	1.18	3.25a	0.79
	Olfactory finesse	**3.25ab**	0.42	**3a**	0.00	**3.75b**	0.42	**3.25ab**	0.42
	Malt smell	2.5a	0.52	3a	0.67	2.5a	0.52	2.5a	0.52
	Hop smell	2.5a	0.84	2.75a	0.48	2.5a	0.52	2.5a	0.53
	Floral smell	**2.5b**	0.52	**2.25b**	0.42	**2.5b**	0.52	**1.25a**	0.42
	Fruity smell	**3.5bc**	0.52	**2.25a**	0.42	**4.25c**	0.92	**2.5ab**	0.52
	Smell of spices	2.75a	0.82	2.25a	0.42	2a	0.00	2a	0.67
	Honey smell	1.5a	0.84	1.75a	0.84	2a	0.79	2a	1.03
	Caramel smell	1.75a	0.48	1.75a	0.84	1.75a	0.84	1.25a	0.42
	Yeast smell	**1.75a**	0.42	**3.25b**	0.42	**1.5a**	0.52	**3.25b**	0.42
	Smoked smell	1.25a	0.42	1.5a	0.84	1.25a	0.42	1.5a	0.84
	Smell of aromatic herbs	1.5a	0.52	1.25a	0.42	1.5a	0.52	1.5a	0.52
Gustative Intensity	Sweetness	2a	0.00	2.5a	0.84	2.25a	0.42	2.5a	0.52
	Bitterness	3.5a	0.52	3.75a	0.48	3.75a	0.48	3.25a	0.48
	Saltiness	2.5a	1.07	2.5a	0.52	3a	0.74	2.5a	0.52
	Acidity	**3a**	0.00	**3a**	0.00	**3.25ab**	0.48	**3.75b**	0.48
	Malty	2.5a	0.52	2.75a	0.84	1.75a	0.84	2.75a	0.42
	Hops	3a	0.67	2.75a	0.42	2.75a	0.84	3.5a	0.52
	Floreal	1.75a	0.48	2a	0.00	1.5a	0.52	1.75a	0.52
	Fruity	2.75a	0.42	2.5a	0.52	3a	1.20	3a	0.67
	Spicy	2.25a	0.82	1.75a	0.79	1.75a	0.84	1.75a	0.84
	Toasted	1.75a	0.84	1.75a	0.84	1.5a	0.52	1.75a	0.84
	Alcohol	3.25a	0.48	3a	0.03	3a	0.03	3.5a	0.52
	Overall quality	**3.75b**	0.88	**3.5b**	0.52	**2.25a**	0.42	**4b**	0.04

The median values within a row (the difference between the storage times) followed by different superscript letters statistically differ ($p < 0.05$); ±sd: standard deviation.

Table S3: Average values for each descriptor rated by consumers. Single and interactive effects of beer sample, gender and age on consumer's acceptability.

	**Appearance liking**	**Odour liking**	**Taste liking**	**Overall liking**
Mean and standard deviation of consumers evaluation				
CHE-3	6.79	6.17	5.74	6.05
±sd	1.39	1.64	1.90	1.68
US05	6.05	6.05	5.64	5.81
±sd	1.82	1.36	1.74	1.58
P4	6.58	6.35	5.77	6.09
±sd	1.36	1.72	2.05	1.72
TA410	6.84	5.81	5.59	5.59
±sd	1.56	1.67	1.88	1.71
p-values of *2-Way ANOVA with interaction*				
Age	0.51	0.25	0.58	0.45
Gender	0.41	0.49	0.24	0.23
Sample	0.23	0.74	0.96	0.90
Age*Gender	0.91	0.17	0.06	0.09
Age*Sample	0.77	0.42	0.73	0.86
Gender*Sample	0.70	0.99	0.97	0.69
p-values of *2-Way ANOVA with no interaction*				
Age	0.30	0.11	0.12	0.11
Gender	0.48	0.97	0.80	0.80
Sample	0.06	0.66	0.99	0.63
p-values of *One-Way ANOVA*				
Age	0.21	0.08	0.09	0.07
Gender	0.25	0.55	0.44	0.37
Sample	0.06	0.64	0.99	0.61

p-values of one-way ANOVA and two-way ANOVA. The two-way ANOVA analysis was applied both considering the linear model and the model with the interaction between factors.
